# Interleukin-32α induces migration of human melanoma cells through downregulation of E-cadherin

**DOI:** 10.18632/oncotarget.11669

**Published:** 2016-08-29

**Authors:** Joohyun Lee, Kyung Eun Kim, Soyoung Cheon, Ju Han Song, Younkyung Houh, Tae Sung Kim, Minchan Gil, Kyung Jin Lee, Seonghan Kim, Daejin Kim, Dae Young Hur, Yoolhee Yang, Sa Ik Bang, Hyun Jeong Park, Daeho Cho

**Affiliations:** ^1^ Department of Life Systems, Sookmyung Women's University, Yongsan-ku, Seoul 140-742, Republic of Korea; ^2^ Department of Cosmetic Sciences, Sookmyung Women's University, Yongsan-ku, Seoul 140-742, Republic of Korea; ^3^ Division of Life Sciences, College of Life Sciences and Biotechnology, Korea University, Seongbuk-gu, Seoul 02841, Republic of Korea; ^4^ Department of Convergence Medicine, Asan Institute for Life Sciences, University of Ulsan College of Medicine, Seoul 138-736, Republic of Korea; ^5^ Department of Anatomy, Inje University College of Medicine, Busan, 614-735, Republic of Korea; ^6^ Department of Plastic Surgery, Samsung Medical Center, Sungkyunkwan University School of Medicine, Gangnam-gu, Seoul 135-710, Republic of Korea; ^7^ Department of Dermatology, Yeouido St. Mary's Hospital, The Catholic University of Korea, Seoul 150-713, Republic of Korea

**Keywords:** interleukin-32, melanoma, migration, Erk1/2, E-cadherin

## Abstract

Interleukin (IL)-32α, the shortest isoform of proinflammatory cytokine IL-32, is associated with various inflammatory diseases and cancers. However, its involvement in human melanoma is not understood. To determine the effect of IL-32α in melanoma, IL-32α levels were examined in human melanoma cell lines that exhibit different migratory abilities. IL-32α levels were higher in human melanoma cell lines with more migratory ability. An IL-32α-overexpressing G361 human melanoma cell line was generated to investigate the effect of IL-32α on melanoma migration. IL-32α-overexpressing G361 cells (G361-IL-32α) exhibit an increased migratory ability compared to vector control cells (G361-vector). To identify factors involved in IL-32α-induced migration, we compared expression of E-cadherin in G361-vector and G361-IL-32α cells. We observed decreased levels of E-cadherin in G361-IL-32α cells, resulting in F-actin polymerization. To further investigate signaling pathways related to IL-32α-induced migration, we treated G361-vector and G361-IL-32α cells with PD98059, a selective MEK inhibitor. Inhibition of Erk1/2 by PD98059 restored E-cadherin expression and decreased IL-32α-induced migration. In addition, cell invasiveness of G361-IL-32α cells was tested using an *in vivo* lung metastasis model. As results, lung metastasis was significantly increased by IL-32α overexpression. Taken together, these data indicate that IL-32α induced human melanoma migration via Erk1/2 activation, which repressed E-cadherin expression. Our findings suggest that IL-32α is a novel regulator of migration in melanoma.

## INTRODUCTION

IL-32 is a recently identified proinflammatory cytokine that was originally known as NK4 transcript [1]. It was identified as one of the genes upregulated in the IL-18-responsive A549 cell line. IL-32 induces various proinflammatory mediators, including IL-1β, IL-6 and tumor necrosis factor alpha (TNF-α) [1, 2]. A large number of studies have investigated the role of IL-32 in inflammatory diseases, including rheumatoid arthritis and inflammatory bowel disease [3, 4]. There are four major IL-32 isoforms that are generated by alternative splicing: IL-32α, IL-32β, IL-32γ, and IL-32δ [1]. Additionally, IL-32ε and IL-32ζ were recently identified as new isoforms of IL-32 [5]. IL-32 isoforms exert their own characteristics. IL-32γ is the longest and most potent IL-32 isoform, whereas IL-32α is the shortest isoform [6]. IL-32β is the predominant isoform expressed in gastric cancer tissue [7] and endothelial cells [8].

Because numerous studies have linked inflammatory cytokines to cancer progression [9], the effect of IL-32 has been investigated in various inflammatory disorders as well as several cancers. IL-32 has pro-cancer effects in most lung cancers and is also used as a prognostic marker for gastric cancer [10, 11]. However, IL-32γ potentiates TNF-α-induced cell growth inhibition [12], suggesting that IL-32 has different roles in different tumor types. Little is known about the effect of IL-32α on cancer progression and studies examining the correlation between cancer and IL-32α are ongoing. Here, to determine the effect of IL-32α on cancer progression, we investigated the pro-cancer effects of IL-32α on melanoma migration.

During melanoma progression, benign melanoma becomes radial-growth phase melanoma (RGP). RGP melanoma can progress into a more aggressive type with metastatic potential, termed vertical phase melanoma (VGP). Transition from RGP to VGP leads to more migratory and invasive abilities, and is associated with alterations of various adhesion molecules, including E-cadherin and N-cadherin [13]. In particular, loss of E-cadherin correlates with poor outcome in patients with cutaneous malignant melanoma [14].

Downregulation of E-cadherin is considered a key feature of cancer progression. During this process, cells lose contact with neighboring cells and acquire migratory ability. E-cadherin is composed of the extracellular region, transmembrane region, and intracellular region, which contains interacting sites with catenins. E-cadherin expression is typically regulated by several factors, including growth factors and cytokines. Hepatocyte growth factor has been shown to downregulate E-cadherin during melanoma development [15]. IL-6 promotes invasion of B16BL6 melanoma cells through E-cadherin downregulation [16]. It is well known that cell morphology is altered if the E-cadherin complex is disrupted during cell migration. After loss of E-cadherin, β-catenin, which anchors the actin cytoskeleton to E-cadherin, is released and phosphorylated. Phosphorylated β-catenin undergoes degradation by ubiquitination [17]. Modified actin cytoskeletons due to β-catenin release contribute to migratory ability [17, 18].

In this study, an IL-32α-overexpressing human melanoma cell line was generated to investigate the effect of IL-32α on melanoma, which has previously not been studied. It was revealed that IL-32α induced melanoma cell migration and reduced E-cadherin expression by Erk1/2 activation. In addition, the selective MEK inhibitor PD98059 restored E-cadherin expression and decreased IL-32α-induced migration. Overall, these findings indicate that IL-32α has pro-cancer effects in human melanoma migration. We suggest that IL-32α might be a therapeutic target in melanoma.

## RESULTS

### IL-32α induces melanoma cell migration

**Figure 1:**
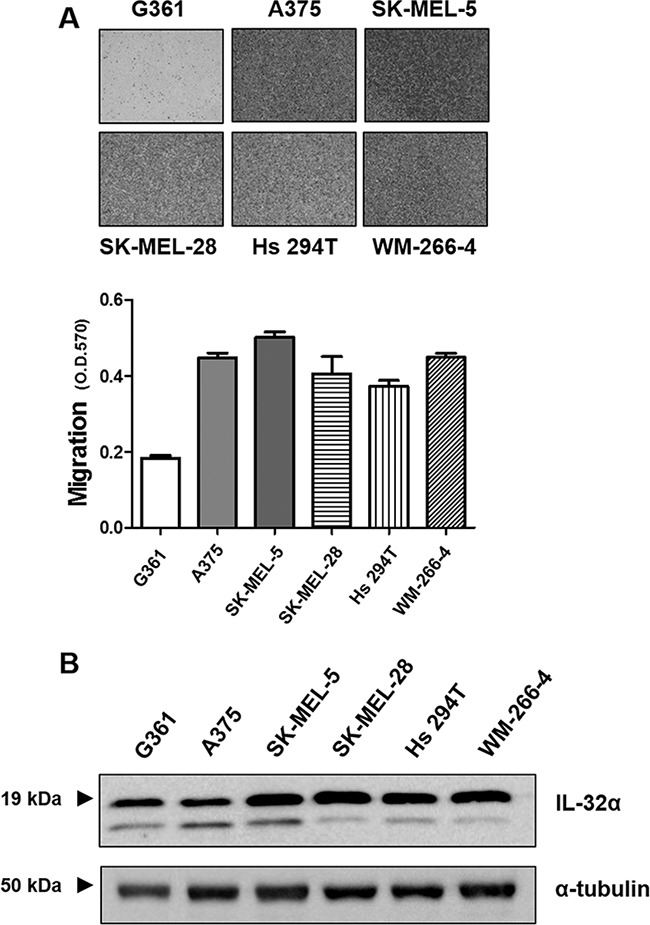
IL-32α expression is higher in migratory human melanoma cell lines **A.** The migratory abilities of human melanoma cell lines G361, A375, SK-MEL-5, SK-MEL-28, Hs 294T, and WM-266-4 were examined. Cells (5×10^4^) were placed in the transwell migration assay upper chambers and DMEM containing 5% FBS was placed in the lower chambers. After incubation for 24 hours, membranes with migrated cells were fixed and stained as described in Materials and Methods. Stained cells were evaluated by microscopy and photographed (magnification, 40×). Membranes were eluted as described and the O.D. at 570 nm was measured. **B.** The human melanoma cell lines were examined for IL-32α expression levels. The lanes of melanoma cell lines are organized in order of migratory ability. Western blotting was performed using the mouse anti-human IL-32α antibody.

To investigate the involvement of IL-32α in melanoma, IL-32α expression levels were compared in various human melanoma cell lines with different migratory abilities. Using a transwell migration assay, we compared the migratory ability of several melanoma cell lines, including G361, A375, SK-MEL-5, SK-MEL-28, Hs 294T, and WM-266-4. G361 exhibited lower migratory ability whereas other human melanoma cell lines exhibited higher migratory abilities. IL-32α expression levels were also evaluated in these human melanoma cell lines and compared to their migratory abilities. SK-MEL-5, SK-MEL-28, Hs 294T, and WM-266-4 cells have high migratory abilities and higher IL-32α expression. G361 cells exhibit lower migratory ability and had the lowest IL-32α expression (Figure 1). This result suggests that there is a positive correlation between melanoma migration and IL-32α expression.

**Figure 2:**
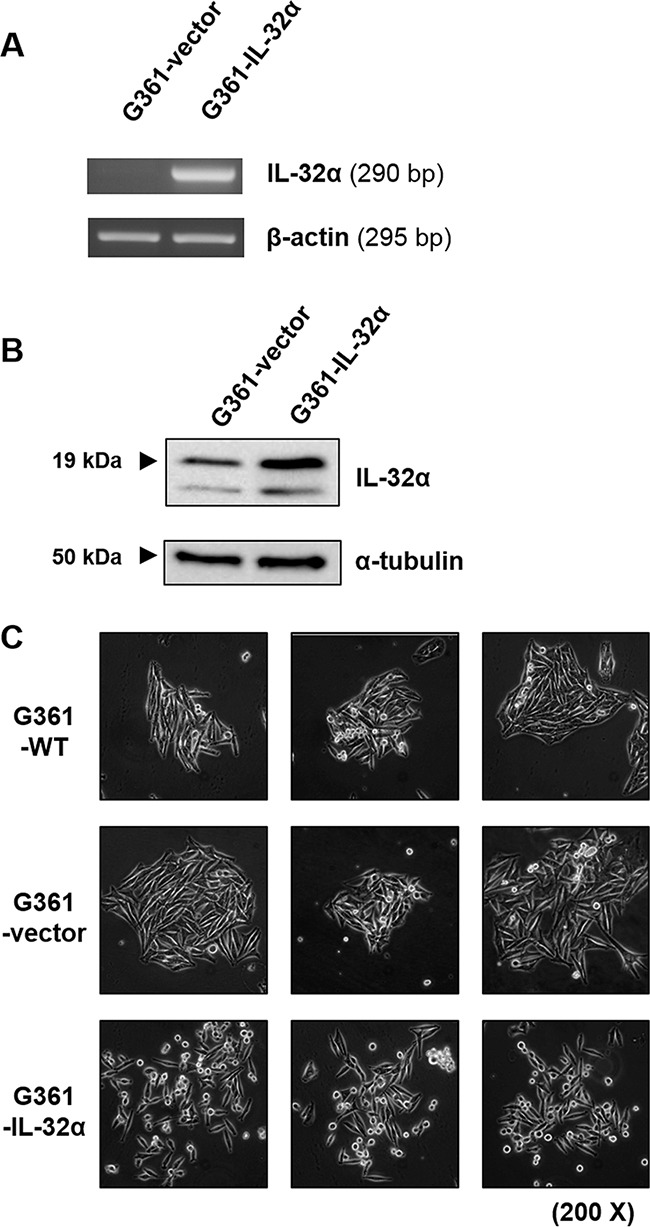
IL-32α-overexpressing G361 cells exhibit a round phenotype **A.** G361 cells were transfected with pcDNA3.1+ or IL-32α/pcDNA3.1+ as described. Total RNA was isolated from transfectants (G361-vector and G361-IL-32α), and the expression level of IL-32α was assessed by RT-PCR. **B.** Transfectants (G361-vector and G361-IL-32α) were lysed and immunoblotted using the mouse anti-human IL-32α antibody to confirm IL-32α overexpression. **C.** G361, G361-vector, and G361-IL-32α cells (5×10^5^) were seeded on culture plates and incubated for 24 hours. Cell morphology was evaluated by microscopy and photographed. Original magnification, 200×. Images are representative of three independent experiments.

To determine the effect of IL-32α on human melanoma migration, an IL-32α-overexpressing stable cell line was generated. G361 cells (with low IL-32α expression) were transfected with pcDNA3.1+/IL-32α or pcDNA3.1+ vectors to generate the stable IL-32α-overexpressing (G361-IL-32α) and vector control cell lines (G361-vector). IL-32α expression was confirmed by RT-PCR and western blotting (Figures 2A and 2B). After transfection, there was a significant morphological difference between G361-vector and G361-IL-32α cells. Interestingly, G361-IL-32α cells acquired a rounder shape and were scattered compared to G361-vector cells (Figure 2C), suggesting that IL-32α induced the migratory ability of cells. Since IL-32α can induce cell apoptosis [19], we performed a TUNEL staining and 7-AAD and Annexin V staining in G361-vector and G361-IL-32α cells to determine whether IL-32α overexpression affects apoptosis in a human melanoma cell line. As a result, there was no difference in the rate of apoptosis between G361-vector and G361-IL-32α cells (Supplementary Figure S1). This result indicates that the overexpression of IL-32α did not affect apoptosis in a human melanoma cell line.

**Figure 3:**
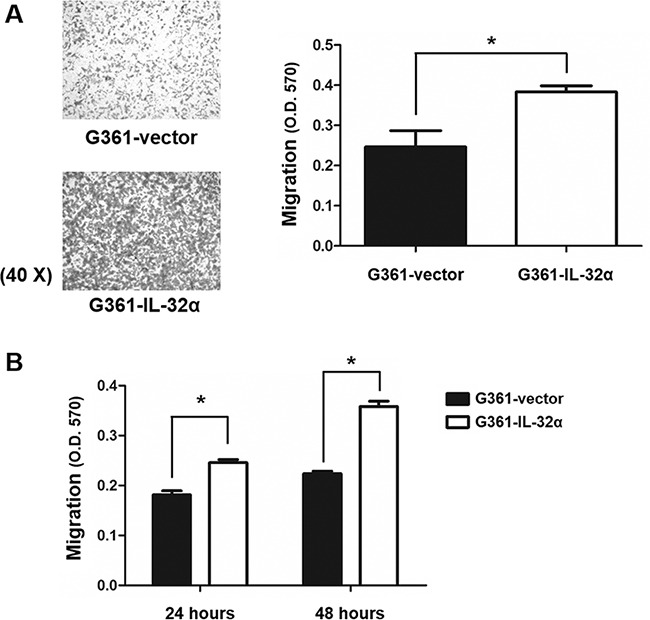
IL-32α overexpression in human melanoma cell lines enhances cell migration **A.** Using transwell chamber assays, the migratory abilities of G361-vector and G361-IL-32α cells were compared. Cells (5×10^4^) were placed in transwell migration upper chambers. DMEM containing 5% FBS was placed in the lower chambers. After incubation for 24 hours, membranes with migrated cells were fixed and stained as described. Stained cells were evaluated by microscopy and photographed (magnification, 40×). Membranes were eluted as described and the O.D. at 570 nm was measured. Data represent the mean ± standard deviation (SD) of one of three independent experiments. *p <0.05 compared to control. **B.** Kinetics of G361-vector and G361-IL-32α cell migration. Cells (5×10^4^) were placed in the upper chamber of transwell chambers. DMEM containing 5% FBS was placed in the lower chamber. Chambers were incubated for 24 and 48 hours. Migrated cells were eluted with 10% acetic acid and the O.D. at 570 nm was measured. All experiments were performed at least three times. A representative experiment of three independent experiments is shown. Data represent the mean ± SD of one of three independent experiments. *p <0.05 compared to the control.

Next, transwell migration assays were performed to compare the migratory ability between G361-vector and G361-IL-32α cells. G361-IL-32α cells exhibited higher migratory ability compared to G361-vector cells (Figure 3). We also treated G361 cells with recombinant human IL-32α (0, 50, and 100 ng/ml) to determine whether exogenous IL-32α treatment could enhance migration of melanoma cells. Exogenous IL-32α treatment did enhance the migratory ability of G361 cells (Supplementary Figure S2A). Taken together, these results suggest that IL-32α induces migration of human melanoma cells.

### IL-32α overexpression induces migration through downregulation of E-cadherin and F-actin polymerization in G361 human melanoma cell lines

**Figure 4:**
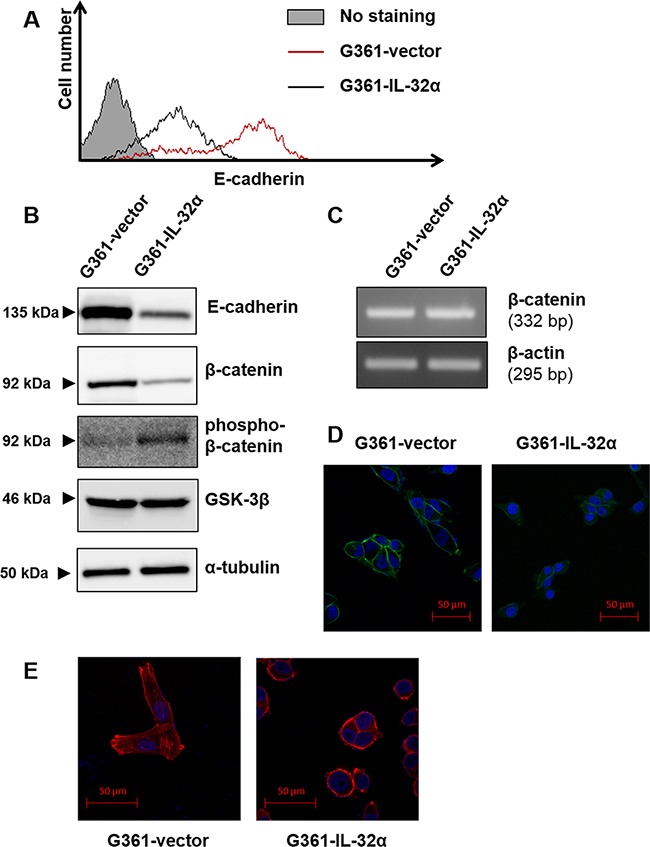
IL-32α overexpression downregulates E-cadherin expression and induces F-actin polymerization **A.** G361-vector and G361-IL-32α cell lines were detached using enzyme-free dissociation buffer. Flow cytometry assays were performed using the PE-conjugated mouse anti-human E-cadherin antibody. **B.** E-cadherin, β-catenin, phospho-β-catenin and GSK-3β expression was evaluated in G361-vector and G361-IL-32α cell lines. **C.** Total RNA was isolated from G361-vector and G361-IL-32α cells. After reverse transcription, PCR was performed with primers for β-catenin or β-actin. **D.** G361-vector and G361-IL-32α cells were attached to coverslips then fixed and permeabilized as described in the Materials and Methods. After permeabilization, the coverslips were blocked with 1% BSA in PBS for 1 hour and incubated at 4°C overnight with rabbit anti-human β-catenin antibody. Coverslips were then incubated with FITC-conjugated goat anti-rabbit IgG antibody. A laser scanning confocal microscope was used for analyses. **E.** G361-vector and G361-IL-32α cells were incubated on coverslips. Cells attached to the coverslips were fixed and permeabilized as mentioned in Materials and Methods. F-actin staining was performed using phalloidin-conjugated Alexa Fluor 647. Confocal microscopy assays were performed as described. These data represent one of three independent experiments.

During melanoma progression, increased migration is accompanied by alterations in adhesion molecule expression [13]. E-cadherin is a major component of adherens junctions and is decreased during melanoma progression [20]. Abnormal expression of E-cadherin deregulates various functions including survival, adhesion, migration, and invasion [21]. To identify factors involved in IL-32α-induced migration, E-cadherin expression was measured in G361-IL-32α cells. We found that IL-32α expression reduced E-cadherin levels in G361 cells (Figures 4A and 4B). Exogenous treatment with recombinant human IL-32α was also able to downregulate E-cadherin expression (Supplementary Figure S2B).

It is well established that disruption of E-cadherin results in β-catenin release. Released β-catenin is phosphorylated by a destruction complex and degraded [18]. Based on these results, we measured β-catenin levels to verify E-cadherin downregulation by IL-32α. The β-catenin levels were dramatically decreased and phospho β-catenin levels were increased in G361-IL-32α cells compared with those in G361-vector cells (Figure 4B). It was revealed that β-catenin transcription was not affected by IL-32α (Figure 4C). These data suggest that downregulation of β-catenin is not mediated at the mRNA level. Since β-catenin is located in multiple sites within the cell, including at the plasma membrane, we performed immunofluorescent staining of β-catenin in G361-vector and G361-IL-32α cells. G361-vector cells exhibited strong β-catenin staining at the plasma membrane whereas G361-IL-32α cells had almost no β-catenin protein at the plasma membrane (Figure 4D). Additionally, there was no change in the GSK-3β level in G361-vector and G361-IL-32α cells (Figure 4B). Collectively, our data suggest that overexpression of IL-32α released β-catenin into the cytoplasm and induced its phosphorylation, which finally leads to degradation of β-catenin.

Along with E-cadherin complex dissociation, cancer cell migration is accompanied by alterations in the actin cytoskeleton, which is involved in cell morphology and migration [22]. Based on the cell morphology shown in Figure 2B, we sought to determine whether F-actin polymerization was increased by IL-32α. Thus, G361-vector and G361-IL-32α cells were stained with phalloidin for F-actin staining. Compared to G361-vector cells, G361-IL-32α cells had increased F-actin polymerization at the cortical region (Figure 4E). These results show that E-cadherin downregulation mediated by IL-32α overexpression exerts F-actin polymerization, thereby leading to cell migration.

### IL-32α-mediated Erk1/2 activation is involved in melanoma migration

**Figure 5:**
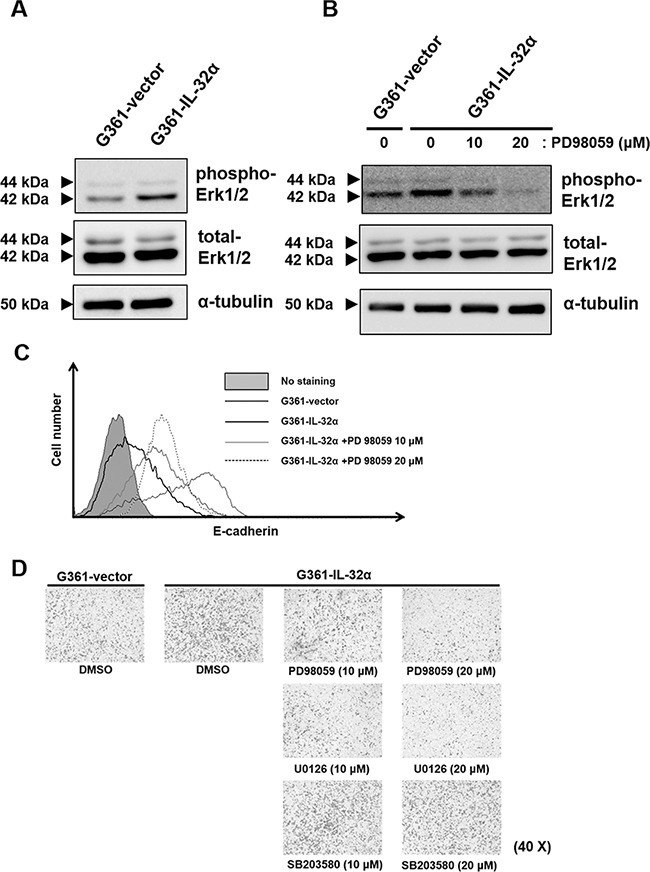
Inhibition of IL-32α-induced Erk1/2 reduces human melanoma cell migration **A.** Erk1/2 phosphorylation levels in G361-vector and G361-IL-32α cells were examined by western blot. Cells were lysed in lysis buffer containing phosphatase inhibitors. Erk1/2 phosphorylation was confirmed using the rabbit anti-human Erk1/2 antibody. **B.** PD98059-mediated inhibition of Erk1/2 phosphorylation was confirmed by western blot. The selective MEK inhibitor PD98059 was added to cells for 24 hours to inhibit Erk1/2 phosphorylation. DMSO was used as a solvent control. After treatment, western blot assays were performed using the rabbit anti-human Erk1/2 antibody. **C.** E-cadherin levels in DMSO- or PD98059-treated cells were examined using flow cytometry. Cells were detached using enzyme-free cell dissociation buffer and incubated with the PE-conjugated mouse anti-human E-cadherin antibody. A representative experiment of three independent experiments is shown. **D.** IL-32α-induced migration was inhibited by Erk1/2 inhibition. G361-IL-32α cells were treated for 24 hours with PD98059, U0126 or SB203580 and collected for transwell migration assays. Cells (5×10^4^) were placed in upper chambers with serum-free DMEM. Lower chambers contained DMEM with 5% FBS. After 24 hours, membranes with migrated cells were fixed and stained as described. Images of membranes were analyzed by microscopy. A representative experiment of three independent experiments is shown.

The Erk1/2 pathway is important for melanoma cell migration [23] and is also involved in actin cytoskeleton rearrangement in melanoma [22]. To investigate whether IL-32α can activate the Erk1/2 pathway, Erk1/2 phosphorylation was measured. Increased Erk1/2 phosphorylation was detected in G361-IL-32α cells compared to G361-vector cells (Figure 5A). We used the selective MEK inhibitor PD98059 to determine whether Erk1/2 is involved in IL-32α-induced migration. Trypan blue staining confirmed that there was no cellular damage caused by PD98059 treatment (data not shown). As shown in Figure 5B, Erk1/2 was effectively inhibited by PD98059 treatment. Subsequently, E-cadherin expression and migration were measured in PD98059-treated and non-treated cells. PD98059 treatment of G361-IL-32α cells restored E-cadherin expression in a dose-dependent manner (Figure 5C). To determine whether Erk1/2 inhibition can repress IL-32α-induced migration, we compared the migratory ability of PD98059-treated G361-IL-32α and non-treated cells. We found that IL-32α-induced migration was dramatically decreased by Erk1/2 inhibition in a PD98059 dose-dependent manner (Figure 5D). Other MAPK inhibitors (U0126 and SB203580) were used to treat G361-IL-32α cells to determine whether IL-32α-induced migration is mediated by Erk1/2 specifically. U0126 is a selective inhibitor of MEK 1/2, which is upstream of MAPK and Erk1/2. SB203580 is a selective inhibitor of p38 MAPK. U0126 treatment in G361-IL-32α cells repressed IL-32α-induced migration, whereas SB203580 treatment did not affect IL-32α-induced migration. Together, these data suggest that IL-32α induces migration via Erk1/2 phosphorylation.

### Overexpression of IL-32α in melanoma cells increases lung metastasis in vivo

**Figure 6:**
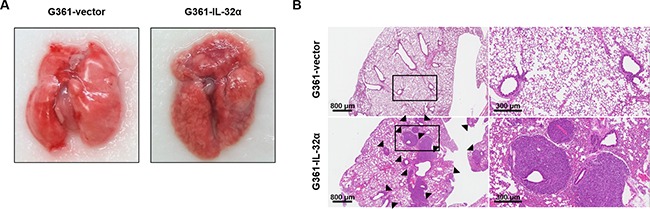
IL-32α expression affects *in vivo* lung metastasis **A.** The effect of IL-32α on invasion was examined using an *in vivo* lung metastasis model. NSG mice (N=5/group) were intravenously injected with 2×10^5^ G361-vector or G361-IL-32α cells in 200 μl PBS. After 6 weeks, mice were sacrificed to observe lung metastasis. Lung tissue samples from both groups were excised and photographed. **B.** Representative images of H&E-stained lung sections. Excised lungs were fixed with 4% paraformaldehyde and embedded in paraffin. Embedded tissue samples were sliced into 8-μm sections and stained with H&E, then photographed with a microscope. Scale bar, 800 μm (left)/300 μm (right). Black arrowheads represent lung metastases.

Based on the effect of IL-32α on melanoma cell migration, we used an *in vivo* lung metastasis model to determine whether IL-32α can affect invasion. G361-vector or G361-IL-32α cells were intravenously injected into NOD.Cg-*Prkdc^scid^ Il2rg^tm1Wjl^*/SzJ (NSG) mice as described in Materials and Methods. After 6 weeks, mice were sacrificed and the lungs were excised. As shown in Figure 6A, lung metastasis was observed in all of the G361-IL-32α cell-injected mice, whereas there was no detectable lung metastasis in any of the G361-vector cell-injected mice. To confirm the presence of lung metastasis, H&E staining was performed using fixed lung tissue with 4% paraformaldehyde. This revealed that mice injected with G361-IL-32α cells exhibited much higher levels of lung metastasis than those injected with G361-vector cells (Figure 6B). Taken together, these results show that IL-32α increased not only migration but also invasion.

## DISCUSSION

Cytokines have a critical role in oncogenic processes, including proliferation, apoptosis, and migration. Especially, pro-inflammatory cytokines such as IL-18, TNF-α, and IL-6 have a positive role in cancer progression, including cancer cell migration. IL-18, a representative pro-inflammatory cytokine, increases cell migration in human gastric cancer and murine melanoma [24, 25]. IL-32 was first reported to be a gene that is upregulated by IL-18. The significant contribution of IL-18 during melanoma progression enabled us to investigate the role of IL-32 in melanoma migration in this study. In this study, we identified IL-32α as a positive factor for melanoma migration. IL-32 is a novel proinflammatory cytokine that stimulates various inflammatory mediators. Many studies have investigated IL-32 in various inflammatory disease as well as in cancer [1, 2]. IL-32 promotes angiogenesis and breast cancer cell proliferation [26, 27]. In the present study, IL-32α was transfected into the human melanoma cell line G361, which has low expression of IL-32α [28]. In the novel IL-32α-overexpressing cell line (G361-IL-32α), migration was significantly increased and E-cadherin expression was downregulated.

During cancer progression, loss of E-cadherin can be regulated in various ways, including transcriptional repression, MAPK signaling, and proteolytic cleavage [21, 29–31]. In most cases, E-cadherin expression is downregulated at the transcriptional level through EMT. Therefore, we analyzed the mRNA levels of transcription factors such as Snail, Slug, and E-cadherin in IL-32α-overexpressing cells. There were no alterations in Snail, Slug, or E-cadherin mRNA expression upon IL-32α overexpression, indicating that IL-32α-induced downregulation of E-cadherin is likely mediated at the protein level (data not shown). The proinflammatory cytokines IFN-γ, and IL-1β increase ADAM10-mediated shedding of E-cadherin by stimulating MAPK signaling in keratinocytes [32]. We examined the expression of several MMPs and their inhibitors, including that of collagenase, gelatinase, stromelysins, and TIMPs (TIMP-1, TIMP-2 and TIMP-4). However, there was no significant difference in the expression of MMPs between G361-vector and G361-IL-32α melanoma cells (Supplementary Figure S3).

Recently, several studies have indicated that IL-32 induces the expression of MMP-2 and MMP-9 in gastric cancer and lung adenocarcinoma [33, 34]. However, IL-32 did not affect MMP-2 and MMP-9 expression in osteosarcoma cells whereas MMP-13 is involved in the IL-32-induced migration [35], suggesting that different types of proteases affects the migratory processes in a cell-type specific manner. It has been well known that several types of proteases are involved in melanoma progression [36]. In addition to MMPs, other enzymes including ADAM families and serine, cysteine proteases also enhance melanoma migration [36, 37]. Therefore, we suggest that other types of proteases could be involved in IL-32α-mediated migratory processes. Further investigation is required to determine the effects of IL-32α on the other types of proteases.

E-cadherin, which is a member of the classical cadherin family, is decreased during melanoma progression. IL-32α expression reduced E-cadherin levels in G361 cells (Figures 4A and 4B). Another classical cadherin, P-cadherin, was also shown to reduce melanoma growth and invasion [38]. However, there were no significant differences between G361-vector and G361-IL-32α cells in the P-cadherin level (data not shown). Therefore, we suggest that IL-32α-induced migration is mediated by E-cadherin, not P-cadherin.

When the E-cadherin adhesion complex dissociates, displaced cytoplasmic partners of E-cadherin repress RhoA and activate Rac1 and Cdc42, which together regulate the actin cytoskeleton and cell migratory ability [17]. Downregulation of E-cadherin results in a loss of cell polarity, which promotes cell migration. As expected, G361-IL-32α cells with decreased E-cadherin expression exhibited a rounded morphology compared to G361-vector cells (Figure 2C). In addition, actin polymerization was increased in G361- IL-32α cells (Figure 4C). Cancer cells usually exhibit a rounded morphology during motility, which is also known as amoeboid movement. It has been known that ROCK activation [39] is involved in amoeboid migration of tumor cells. We treated G361-IL-32α cells with ROCK inhibitor Y27632 to determine whether inhibition of ROCK can affect IL-32α-mediated migration. The increased migration mediated by IL-32α was diminished by ROCK inhibitor treatment (Supplementary Figure S4). This suggested that the round morphology and increased migration caused by IL-32α is mediated by ROCK activation.

Based on the results of our *in vitro* experiments, we used a lung metastasis mouse model to examine whether IL-32α can affect invasion ability *in vivo*. Because no mouse homolog of IL-32 has been discovered, G361-vector and G361-IL-32α cells were intravenously injected into NSG mice, which are depleted of immune cells including B, T, and NK cells. As shown in Figure 6, the increased lung metastasis *in vivo* revealed that expression of IL-32α can affect the ability of cells to invade. In addition, G361-vector or G361-IL-32α cells were subcutaneously injected into SCID mice as mentioned in Supplementary Materials and Methods. As a result, tumors had developed in mice injected with G361-IL-32α. However, most mice injected with G361-vector cells showed a low incidence (Supplementary Table S1). Collectively, we conclude that IL-32α affects melanoma cell invasion *in vivo* in addition to migration *in vitro*, and therefore has tumorigenic properties in melanoma cells.

This study examined the effect of IL-32α on human melanoma cells and found that IL-32α positively contributes to melanoma progression. Taken together, our data show that IL-32α activates Erk1/2, which leads to E-cadherin disruption and F-actin polymerization in human melanoma cells, thereby resulting in increased migration. In conclusion, we propose that IL-32α is involved in the progression of melanoma and could be a novel regulator of migration or a therapeutic target for this disease.

## MATERIALS AND METHODS

### Cell culture and transfections

The human melanoma cell lines G361, A375, SK-MEL-5, SK-MEL-28, Hs 294T and WM-266-4 were purchased from ATCC or KCLB and cultured in Dulbecco's Modified Eagle media (DMEM) with 10% fetal bovine serum (FBS). Cells were maintained at 37°C in a 5% CO_2_ humidified incubator. Using the Neon® Transfection System (Life Technologies), G361 cells were transfected with the IL-32α/pcDNA3.1+ vector or pcDNA3.1+ vector (as vector control). Transfections were performed at 1000 V/40 ms/1 pulse. Transfectants were cultured in DMEM with 10% FBS containing 500 μg/ml G418 (Clontech, Mountain View, CA) for selection. IL-32α overexpression was confirmed by western blot.

### RT-PCR

Total RNA was extracted from G361-vector and G361-IL-32α cells using Trizol (Invitrogen) according to the manufacturer's instructions. The cDNAs were used as a template for PCR amplification with primers, whose sequences were as follows: β-actin, 5′-TCACCCACACTGTGCCCATCTACG-3′ (forward) and 5′-CAGCGGAACCGCTCATTGCCAATG-3′ (reverse); IL-32α, 5′-CTGAAGGCCCGAATGCACCA-3′ (forward) and 5′-CCGTAGGACTTGTCACAAAA-3′ (reverse) β-catenin, 5′-TGCCAAGTGGGTGGTATAG AG-3′ (forward) and 5′-CGCTGGGTATCCTGATGTGC-3′ (reverse). The cycling conditions were as follows: (β-actin and β-catenin) 94°C for 30 seconds, 60°C for 30 seconds, and 72°C for 30 seconds for 25 cycles; (IL-32α) 94°C for 20 seconds, 56°C for 10 seconds, and 70°C for 40 seconds for 30 cycles.

### Western blot analyses

Cells were washed with cold phosphate buffered saline (PBS) and lysed in Pro-Prep solution (Intron, Korea) containing a phosphatase inhibitor cocktail (Sigma, Saint Louis, MO). After lysis, protein quantification was performed using Bradford assays (Biorad, Hercules, CA). Equal amounts of protein were resolved in sodium dodecyl sulfate (SDS)-polyacrylamide gels. Separated proteins were transferred to polyvinylidene fluoride (PVDF) membranes (Biorad, Hercules, CA). Subsequently, membranes were incubated for 30 minutes in blocking solution (5% nonfat milk), and incubated overnight at 4°C with mouse anti-human IL-32α (Biolegend, San Diego, CA), mouse anti-human α-tubulin (Sigma, Saint Louis, MO), rabbit anti-human β-catenin, rabbit anti-huamn phospho-β-catenin, rabbit anti-human GSK-3β, rabbit anti-human E-cadherin, rabbit anti-human phospho-p44/42 MAPK or rabbit anti-human total p44/42 MAPK (Cell Signaling, Danvers, MA) antibodies (1:1000). After incubation with the primary antibody, membranes were washed three times with PBS containing 0.1% Tween 20 (Merck, Germany) and incubated for 1 hour at room temperature with horseradish peroxidase (HRP)-conjugated secondary antibodies (Jackson Laboratory, West Grove, PA). Target proteins were visualized using an ECL system (Amersham Biosciences, UK) and LAS3000 (Fuji Film).

### Transwell migration assays

Migration assays were performed using 24-well Transwell® culture chambers (Costar, Cambridge, MA). Lower chambers were filled with DMEM containing 5% FBS. Equal numbers (5×10^4^) of human melanoma cell lines (G361, A375, SK-MEL-5, SK-MEL-28, Hs 294T and WM-266-4) or transfectants (G361-vector and G361-IL-32α) were added to the upper insert with serum-free DMEM. Transwell chambers were incubated at 37°C in a 5% CO_2_ humidified incubator for 24 hours. After incubation, migrated cells were fixed with methanol and stained with crystal violet solution. After imaging, the stained cells were eluted in 10% acetic acid and optical density (O.D.) values at 570 nm were measured using an ELISA reader (Molecular Devices, Sunnyvale, CA). The migratory ability of cells was assessed in triplicate wells.

### Flow cytometry assays

Cells were detached with enzyme-free cell dissociation buffer (Life Technologies, UK). Cells (5×10^5^) were washed twice with PBS and incubated for 30 minutes on ice with the PE-conjugated mouse anti-human E-cadherin antibody (R&D Systems, Minneapolis, MN). Sequentially, cells were washed twice with PBS and resuspended in equal volumes of PBS. Analyses were performed using a FACS Calibur (BD).

### Confocal microscope assays

Cells (1×10^5^) were seeded on coverslips for attachment. Next, the coverslips were fixed for 10 minutes at room temperature with 4% paraformaldehyde in PBS. Each coverslip was incubated with 0.1% Triton X-100 in PBS for 5 minutes and washed three times with PBS. Coverslips were incubated for 20 minutes at room temperature with Alexa Fluor 647 phalloidin (Molecular probes, Eugene, OR) diluted in PBS containing 1% bovine serum albumin (BSA). For the detection of β-catenin, cells on coverslips and fixed with prewarmed 4% paraformaldehyde in PBS for 15 minutes. The fixed cells were washed with PBS and incubated with 0.2% Triton X-100 for 30 minutes. After permeabilization, the coverslips were incubated with 1% BSA in PBS for 1 hour and incubated at 4°C overnight with the rabbit anti-human β-catenin (1:200). The coverslips were washed twice with 1% BSA in PBS and incubated with FITC conjugated goat anti-rabbit IgG antibody for 30 minutes. The coverslips were then washed three times and mounted. VECTASHIELD mounting media with DAPI (Vector Laboratories, Burlingame, CA) was used to mount the coverslips. A laser scanning confocal microscope (Zeiss) was used for analyses.

### Inhibitor assays

The selective MEK inhibitor PD98059 (Merck, Germany) was used to inhibit Erk1/2 phosphorylation. G361-vector and G361-IL-32α cells were seeded in 10 cm2 plates, and PD98059 (10 μM or 20 μM) was added once for 24 hours. DMSO was used as a solvent control. Inhibition of Erk1/2 phosphorylation was confirmed using western blot assays. After PD98059 treatment, E-cadherin levels were examined using flow cytometry assays. Briefly, to detect E-cadherin expression, cells were detached with enzyme-free cell dissociation buffer. Cells (5×10^5^) were incubated with the PE-conjugated mouse anti-human E-cadherin antibody. E-cadherin expression was examined using flow cytometry assays. To measure migration, lower chambers were filled with DMEM containing 5% FBS. DMSO- or PD98059-treated cells (5×10^4^) were added to the upper insert. After 24 hours, migrated cells were fixed with methanol and stained with crystal violet solution. Stained cells were photographed.

The cells were also treated once for 24 hours with the selective MEK1/2 inhibitor U0126 or the selective p38 MAPK inhibitor SB203580 (Merk, 10 μM or 20 μM). After U0126 or SB203580 treatment, transwell migration assays were performed as described above.

### In vivo lung metastasis assay

NOD.Cg-*Prkdc^scid^ Il2rg^tm1Wjl^*/SzJ (NSG) mice were obtained from The Jackson Laboratory. These mice were housed in a specific pathogen-free facility at Korea University. The experiment was performed according to the guidelines of the Korea University Institutional Animal Care and Use Committee. To establish an experimental lung metastasis model, 7- to 8-week-old NSG mice were randomly assigned to two groups (5 mice per group) and intravenously injected with 2×10^5^ G361-vector or G361-IL-32α cells 200 μl PBS buffer. Six weeks later, mice were sacrificed and the excised lungs were fixed with 4% paraformaldehyde.

For evaluation of lung metastasis, fixed lung tissue samples were embedded in paraffin. The paraffin-embedded samples were cut into 8-μm sections. Each section was stained with hematoxylin and eosin (H&E). H&E staining results were photographed using a microscope.

### Statistical analysis

Statistical analyses were performed using unpaired Student's *t*-tests. Mean differences were considered significant when *p* <0.05.

## SUPPLEMENTARY MATERIALS FIGURES AND TABLE



## References

[1] Kim SH, Han SY, Azam T, Yoon DY, Dinarello CA (2005). Interleukin-32: a cytokine and inducer of TNFalpha. Immunity.

[2] Joosten LA, Netea MG, Kim SH, Yoon DY, Oppers-Walgreen B, Radstake TR, Barrera P, van de Loo FA, Dinarello CA, van den Berg WB (2006). IL-32 a proinflammatory cytokine in rheumatoid arthritis. Proceedings of the National Academy of Sciences of the United States of America.

[3] Heinhuis B, Koenders MI, van Riel PL, van de Loo FA, Dinarello CA, Netea MG, van den Berg WB, Joosten LA (2011). Tumour necrosis factor alpha-driven IL-32 expression in rheumatoid arthritis synovial tissue amplifies an inflammatory cascade. Annals of the rheumatic diseases.

[4] Shioya M, Nishida A, Yagi Y, Ogawa A, Tsujikawa T, Kim-Mitsuyama S, Takayanagi A, Shimizu N, Fujiyama Y, Andoh A (2007). Epithelial overexpression of interleukin-32alpha in inflammatory bowel disease. Clinical and experimental immunology.

[5] Goda C, Kanaji T, Kanaji S, Tanaka G, Arima K, Ohno S, Izuhara K (2006). Involvement of IL-32 in activation-induced cell death in T cells. International immunology.

[6] Choi JD, Bae SY, Hong JW, Azam T, Dinarello CA, Her E, Choi WS, Kim BK, Lee CK, Yoon DY, Kim SJ, Kim SH (2009). Identification of the most active interleukin-32 isoform. Immunology.

[7] Sakitani K, Hirata Y, Hayakawa Y, Serizawa T, Nakata W, Takahashi R, Kinoshita H, Sakamoto K, Nakagawa H, Akanuma M, Yoshida H, Maeda S, Koike K (2012). Role of interleukin-32 in Helicobacter pylori-induced gastric inflammation. Infect Immun.

[8] Heinhuis B, Koenders MI, van de Loo FA, Netea MG, van den Berg WB, Joosten LA (2011). Inflammation-dependent secretion and splicing of IL-32{gamma} in rheumatoid arthritis. Proceedings of the National Academy of Sciences of the United States of America.

[9] Lu H, Ouyang W, Huang C (2006). Inflammation, a key event in cancer development. Molecular cancer research.

[10] Sorrentino C, Di Carlo E (2009). Expression of IL-32 in human lung cancer is related to the histotype and metastatic phenotype. American journal of respiratory and critical care medicine.

[11] Ishigami S, Arigami T, Uchikado Y, Setoyama T, Kita Y, Sasaki K, Okumura H, Kurahara H, Kijima Y, Harada A, Ueno S, Natsugoe S (2013). IL-32 expression is an independent prognostic marker for gastric cancer. Medical oncology.

[12] Marcondes AM, Mhyre AJ, Stirewalt DL, Kim SH, Dinarello CA, Deeg HJ (2008). Dysregulation of IL-32 in myelodysplastic syndrome and chronic myelomonocytic leukemia modulates apoptosis and impairs NK function. Proceedings of the National Academy of Sciences of the United States of America.

[13] Bar-Eli M (2001). Gene regulation in melanoma progression by the AP-2 transcription factor. Pigment cell research.

[14] Kreizenbeck GM, Berger AJ, Subtil A, Rimm DL, Gould Rothberg BE (2008). Prognostic significance of cadherin-based adhesion molecules in cutaneous malignant melanoma. Cancer epidemiology biomarkers & prevention.

[15] Li G, Schaider H, Satyamoorthy K, Hanakawa Y, Hashimoto K, Herlyn M (2001). Downregulation of E-cadherin and Desmoglein 1 by autocrine hepatocyte growth factor during melanoma development. Oncogene.

[16] Kushiro K, Chu RA, Verma A, Nunez NP (2012). Adipocytes Promote B16BL6 Melanoma Cell Invasion and the Epithelial-to-Mesenchymal Transition. Cancer microenvironment.

[17] Christofori G (2006). New signals from the invasive front. Nature.

[18] Cavallaro U, Christofori G (2004). Cell adhesion and signalling by cadherins and Ig-CAMs in cancer. Nature reviews Cancer.

[19] Meyer N, Zimmermann M, Burgler S, Bassin C, Woehrl S, Moritz K, Rhyner C, Indermitte P, Schmid-Grendelmeier P, Akdis M, Menz G, Akdis CA (2010). IL-32 is expressed by human primary keratinocytes and modulates keratinocyte apoptosis in atopic dermatitis. J Allergy Clin Immunol.

[20] John JK, Paraiso KH, Rebecca VW, Cantini LP, Abel EV, Pagano N, Meggers E, Mathew R, Krepler C, Izumi V, Fang B, Koomen JM, Messina JL, Herlyn M, Smalley KS (2012). GSK3beta inhibition blocks melanoma cell/host interactions by downregulating N-cadherin expression and decreasing FAK phosphorylation. The Journal of investigative dermatology.

[21] Carneiro P, Fernandes MS, Figueiredo J, Caldeira J, Carvalho J, Pinheiro H, Leite M, Melo S, Oliveira P, Simoes-Correia J, Oliveira MJ, Carneiro F, Figueiredo C, Paredes J, Oliveira C, Seruca R (2012). E-cadherin dysfunction in gastric cancer--cellular consequences clinical applications and open questions. FEBS letters.

[22] Klein RM, Spofford LS, Abel EV, Ortiz A, Aplin AE (2008). B-RAF regulation of Rnd3 participates in actin cytoskeletal and focal adhesion organization. Molecular biology of the cell.

[23] Gray-Schopfer V, Wellbrock C, Marais R (2007). Melanoma biology and new targeted therapy. Nature.

[24] Kim KE, Song H, Kim TS, Yoon D, Kim CW, Bang SI, Hur DY, Park H, Cho DH (2007). Interleukin-18 is a critical factor for vascular endothelial growth factor-enhanced migration in human gastric cancer cell lines. Oncogene.

[25] Jung MK, Song HK, Kim KE, Hur DY, Kim T, Bang S, Park H, Cho DH (2006). IL-18 enhances the migration ability of murine melanoma cells through the generation of ROI and the MAPK pathway. Immunology letters.

[26] Nold-Petry CA, Rudloff I, Baumer Y, Ruvo M, Marasco D, Botti P, Farkas L, Cho SX, Zepp JA, Azam T, Dinkel H, Palmer BE, Boisvert WA (2014). IL-32 promotes angiogenesis. Journal of immunology.

[27] Park JS, Choi SY, Lee JH, Lee M, Nam ES, Jeong AL, Lee S, Han S, Lee MS, Lim JS, Yoon do Y, Kwon Y, Yang Y (2013). Interleukin-32beta stimulates migration of MDA-MB-231 and MCF-7cells via the VEGF-STAT3 signaling pathway. Cellular oncology.

[28] Joosten LA, Heinhuis B, Netea MG, Dinarello CA (2013). Novel insights into the biology of interleukin-32. Cellular and molecular life sciences.

[29] Lee DJ, Kang DH, Choi M, Choi YJ, Lee JY, Park JH, Park YJ, Lee KW, Kang SW (2013). Peroxiredoxin-2 represses melanoma metastasis by increasing E-Cadherin/beta-Catenin complexes in adherens junctions. Cancer research.

[30] David JM, Rajasekaran AK (2012). Dishonorable discharge: the oncogenic roles of cleaved E-cadherin fragments. Cancer research.

[31] Symowicz J, Adley BP, Gleason KJ, Johnson JJ, Ghosh S, Fishman DA, Hudson LG, Stack MS (2007). Engagement of collagen-binding integrins promotes matrix metalloproteinase-9-dependent E-cadherin ectodomain shedding in ovarian carcinoma cells. Cancer research.

[32] Maretzky T, Scholz F, Koten B, Proksch E, Saftig P, Reiss K (2008). ADAM10-mediated E-cadherin release is regulated by proinflammatory cytokines and modulates keratinocyte cohesion in eczematous dermatitis. The Journal of investigative dermatology.

[33] Tsai CY, Wang CS, Tsai MM, Chi HC, Cheng WL, Tseng YH, Chen CY, Lin CD, Wu JI, Wang LH, Lin KH (2014). Interleukin-32 increases human gastric cancer cell invasion associated with tumor progression and metastasis. Clinical cancer research.

[34] Zeng Q, Li S, Zhou Y, Ou W, Cai X, Zhang L, Huang W, Huang L, Wang Q (2014). Interleukin-32 contributes to invasion and metastasis of primary lung adenocarcinoma via NF-kappaB induced matrix metalloproteinases 2 and 9 expression. Cytokine.

[35] Zhou Y, Hu Z, Li N, Jiang R (2015). Interleukin-32 stimulates osteosarcoma cell invasion and motility via AKT pathway-mediated MMP-13 expression. Int J Mol Med.

[36] Moro N, Mauch C, Zigrino P (2014). Metalloproteinases in melanoma. Eur J Cell Biol.

[37] Gangemi R, Amaro A, Gino A, Barisione G, Fabbi M, Pfeffer U, Brizzolara A, Queirolo P, Salvi S, Boccardo S, Gualco M, Spagnolo F, Jager MJ, Mosci C, Rossello A, Ferrini S (2014). ADAM10 correlates with uveal melanoma metastasis and promotes in vitro invasion. Pigment Cell Melanoma Res.

[38] Jacobs K, Van Gele M, Forsyth R, Brochez L, Vanhoecke B, De Wever O, Bracke M (2010). P-cadherin counteracts myosin II-B function: implications in melanoma progression. Molecular cancer.

[39] Saito K, Ozawa Y, Hibino K, Ohta Y (2012). FilGAP a Rho/Rho-associated protein kinase-regulated GTPase-activating protein for Rac controls tumor cell migration. Molecular biology of the cell.

