# Supplementary material for: Interleukin-32α induces migration of human melanoma cells through downregulation of E-cadherin
Source: Oncotarget. 2016 Aug 29;7(40):65825–36. doi: 10.18632/oncotarget.11669 (PMC5323195; doi:10.18632/oncotarget.11669)
Supplement: Supplementary file 1 [file oncotarget-07-65825-s001.pdf]

Interleukin-32α induces migration of human melanoma cells through downregulation of E-cadherin

SUPPLEMENTARY MATERIALS AND METHODS

Transwell migration assays

Recombinant human IL-32α (0, 50, and 100 ng/ml) was purchased from R&D systems and treated to G361 cells for 24 hours. After incubation, migration assays were performed using 24-well Transwell® culture chambers (Costar, Cambridge, MA). Lower chambers were filled with DMEM containing 5% FBS. Equal numbers (5×10^4) of cells were placed to the upper insert with serum-free DMEM. Transwell chambers were incubated for 24 hours. After incubation, migrated cells were fixed with methanol and stained with crystal violet solution.

To perform ROCK inhibitor assay, G361-IL-32α cells were treated with ROCK inhibitor Y27632 (Sigma, 0 and 10 μM) for 24 hours. G361-vector and G361-IL-32α cells treated with Y27632 or untreated were detached with enzyme-free dissociation buffer and placed into transwell migration chambers. Then, transwell migration assay was performed as described in the Materials and Methods.

Flow cytometry assays

Cells treated with recombinant human IL-32α (0, 50, and 100 ng/ml) for 24hours were detached with enzyme-free cell dissociation buffer (Life Technologies, UK). Cells (5×10^5) were washed with PBS and incubated for 30 minutes on ice with the PE-conjugated mouse anti-human E-cadherin antibody (R&D Systems, Minneapolis, MN). Then, cells were washed twice with PBS and flow cytometry analysis was performed using a FACS Calibur (BD).

To detect apoptotic cells in G361-vector and G361-IL-32α cells, cells (5×10^5) were washed with PBS and stained with FITC conjugated Annexin V and 7-AAD (BD). After staining, stained cells were analyzed with flow cytometry analysis.

TUNEL assay

A TUNEL (terminal deoxynucleotidyl transferase-mediated d-UTP biotin nick end labeling) assay was performed using the DeadEnd™ Colorimetric TUNEL System (Promega, WI, USA) according to the manufacturer's protocol. Briefly, cells on poly-L-lysine-coated slides were immersed into 4% paraformaldehyde for 25 minutes and washed with PBS. The slides were permeabilized, and equilibrated with Equilibration Buffer. TdT reaction mix solution was added to the cells on the slides for 60 minutes at 37°C. After incubation, reaction was stopped with 2X SSC solution and washed with PBS. Then, slides were immersed in 0.3% hydrogen peroxide for 5 minutes. Streptavidin HRP solution was added to slides for 30 minutes. The slides were stained with DAB and mounted. After staining, stained slides were examined by microscope and photographed.

Human MMP antibody array

G361-vector and G361-IL-32α cells (5×10^5) were incubated in 6–well plates for overnight in DMEM with 1% FBS. After incubation, cell culture supernatant from G361-vector and G361-IL-32α cells were collected for analysis of human MMP using human MMP antibody array kit (abcam). The human MMP antibody array was performed according to the manufacturer's protocol. Briefly, membranes were incubated with 1X Blocking Buffer at room temperature for 30 minutes. Cell culture supernatants were treated to membrane for overnight at 4°C. After the overnight incubation, membranes were washed with wash buffer. A cocktail of biotin-conjugated antibodies was added to membrane. HRP-conjugated streptavidin solution was added and chemiluminescence detection was performed using an ECL system and LAS3000 (Fuji Film). To analyze array data, comparison of signal intensities for individual spots was performed using TotalLab (nonlinear dynamics).

Animal studies

The experiments were performed according to the guidelines of Korea University Institutional Animal Care and Use Committee. Eight-week-old female severe combined immunodeficient mice (NOD.CB17-*Prkdc^scid^*/Arc, Animal Resources Centre, Canning Vale, Australia) were housed in a specific pathogen-free facility at the Korea University and randomly grouped before initiation of experiments. G361-vector and G361-IL-32α cells (1×10^6/mice) were inoculated subcutaneously between the scapula of each scid mice group (n=7 and n=8, respectively). The tumor incidence was assessed for 7 weeks.

SUPPLEMENTARY FIGURES AND TABLE

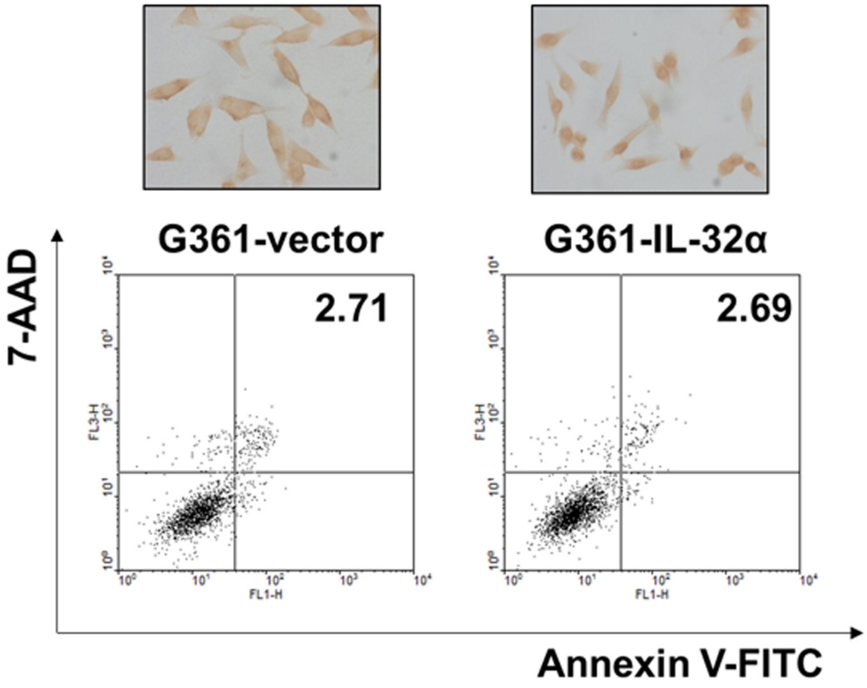

Supplementary Figure S1: Overexpression of IL-32α did not affect apoptosis in a human melanoma cell line. TUNEL assay was performed as described in supplementary materials and methods. G361-vector and G361-IL-32α cells were added to poly-L-lysine-coated slides and fixed and permeabilized. Reaction was performed using TdT reaction mix solution. After incubation, reaction was stopped and washed with PBS. Then, 0.3% hydrogen peroxide was added to slides for 5 minutes. Streptavidin HRP solution was applied to slides for 30 minutes. The slides were stained with DAB and mounted. After staining, stained slides were examined by microscope and photographed. For flow cytometry analysis, G361-vector and G361-IL-32α cells (5×10^5) were washed with PBS and stained with FITC conjugated Annexin V and 7-AAD. After staining, cells were analyzed with FACS calibur.

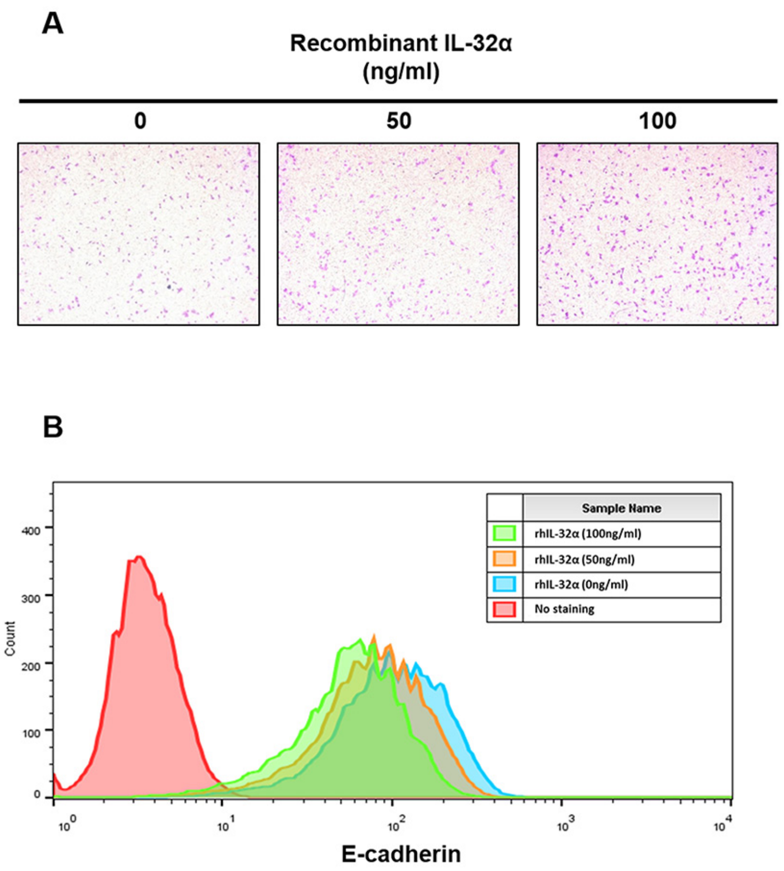

Supplementary Figure S2: Recombinant human IL-32α downregulates E-cadherin expression and enhances G361 cell migration. A. Recombinant human IL-32α (0, 50, and 100 ng/ml) was added to G361 cells. After a 24-hour incubation, cells were collected to compare the migration ability using a transwell migration assay. Cells (5×10^4) were placed in the transwell upper chambers and DMEM containing 5% FBS was placed in the lower chambers. After incubation for 24 hours, membranes were fixed and stained. Stained cells were evaluated by microscopy and photographed (magnification, 40×). **B.** G361 cells incubated with or without recombinant human IL-32α (0, 50 and 100 ng/ml) were collected to measure E-cadherin expression using flow cytometry. Cells were detached using enzyme-free dissociation buffer. Flow cytometry assays were performed using the PE-conjugated mouse anti-human E-cadherin antibody.

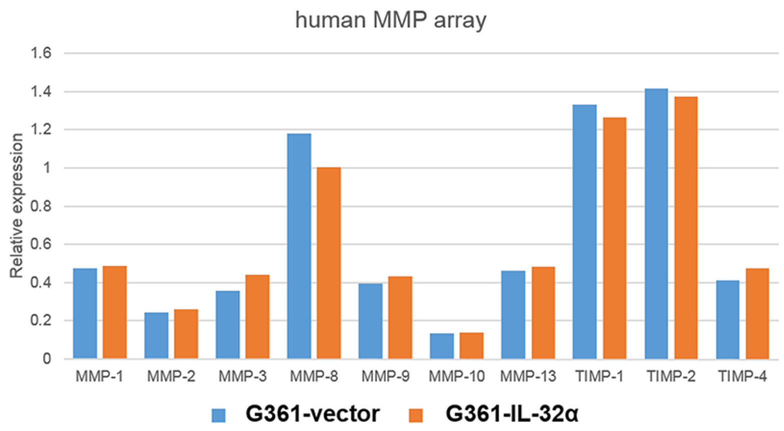

Supplementary Figure S3: IL-32α overexpression has no effect on MMP expression. G361-vector and G361-IL-32α cells (5×10^5) were placed in 6–well plates and incubated overnight in DMEM with 1% FBS. The expression of MMPs and TIMPs in the culture supernatant was measured using a human MMP antibody array kit which includes 10 targets, according to the manufacturer's protocol. Briefly, the antibody array membrane was blocked by blocking buffer. Supernatants from G361-vector and G361-IL-32α cells were incubated with the membrane overnight at 4°C. After incubation, membranes were washed with wash buffer and a cocktail of biotin-conjugated antibodies was added to membrane. HRP-conjugated streptavidin solution was added and chemiluminescence detection was performed using an ECL method.

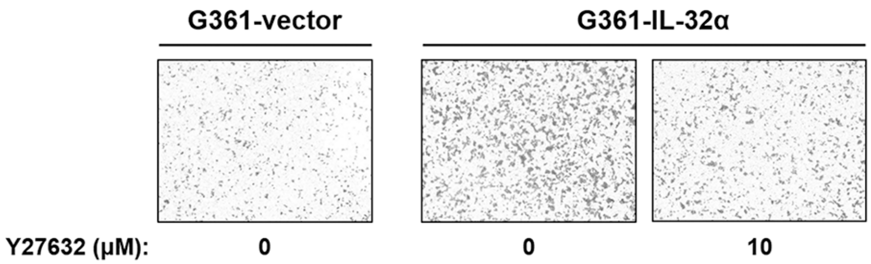

Supplementary Figure S4: IL-32α-mediated migration is reduced by ROCK inhibitor treatment. G361-IL-32α cells were treated with ROCK inhibitor Y27632 (0 and 10 μM) for 24 hours. G361-vector and G361-IL-32α cells treated with Y27632 or untreated were detached with enzyme-free dissociation buffer and placed into transwell migration chambers. The transwell migration assay was performed as described in the Materials and Methods. Briefly, cells (5×10^4) were placed in the upper transwell chambers. DMEM containing 5% FBS was placed in the lower chamber. After 24 hours, upper chambers were fixed with methanol and stained with crystal violet solution.

Supplementary Table S1: The incidence of tumor development in primary region after subcutaneous injection of G361 cells

	G361-vector	G361-IL-32
Incidence (%)	2/7 (28.6%)	8/8 (100.0%)
